# Individual and community-level factors associated with lifetime number of sexual partners among women aged 15–49 in Eswatini

**DOI:** 10.1371/journal.pone.0246100

**Published:** 2021-01-26

**Authors:** Maswati S. Simelane, Kerry Vermaak, Eugene Zwane, Sdumo Masango

**Affiliations:** 1 Department of Statistics and Demography, Faculty of Social Sciences, University of Eswatini, Kwaluseni, Eswatini; 2 The School of Built Environment and Development Studies, University of KwaZulu-Natal, Durban, South Africa; Beth Israel Deaconess Medical Center/Harvard Medical School, UNITED STATES

## Abstract

**Introduction:**

Understanding the risk factors for behavioral patterns in sexual relationships play a significant role in the reduction of the transmission of HIV/AIDS and other sexually transmitted infections.

**Objective:**

To investigate individual and community level factors on the lifetime number of sexual partners of women in Eswatini

**Material and methods:**

The study was a secondary cross-sectional analysis of the 2014 Eswatini Multiple Indicator Cluster Survey (MICS). A total of 2,832 women aged 15–49 years were asked in total, how many different people have you had sexual intercourse in your lifetime. The multilevel negative binomial regression model was used to analyze the data.

**Results:**

The overall mean number of lifetime sexual partners was 2.78 (95% CI: 2.66, 2.91) in 2014. Compared to women aged 15–19, those aged 20 years and older, formerly married or never married reported more lifetime sexual partners compared to currently married women. Those that were aged 15 years and older at sexual debut reported fewer lifetime sexual partners compared to those that were aged less than 15 years. Compared to women that used a condom at last sexual intercourse, those that did not use a condom at last sexual encounter reported fewer lifetime sexual partners. Relative to women that lived with their sons and daughters, those that did not live with their sons and daughters reported more lifetime sexual partners. Women that lived in the Shiselweni and Lubombo regions reported fewer lifetime sexual partners compared to those residents in the Hhohho region.

**Conclusion:**

Overall, lifetime sexual partners in Eswatini was significantly associated with individual characteristics and is unique across regions. Programs that aim to elucidate the factors associated with incident HIV infections among women in Eswatini should focus on individual and community-level factors that are associated with multiple sexual partnerships, which in turn might increase the risk of HIV exposure.

## Introduction

Eswatini has a generalized epidemic with an HIV prevalence of 26% in 2007 [1] and 27% among adults aged 15–49 years, 20.4% among males, and 32.5% among females in 2016 [2]. The HIV incidence was estimated at 2.4% (95% CI: 2.1, 2.8) in 2016, higher among women 3.1% (95% CI: 2.6, 3.7) versus 1.7% (95% CI: 1.3, 2.1) in men [2, 3].

To reduce the incidence of HIV infection, a combination of approaches have been implemented in Eswatini. In 2011, a substantial scale-up to access antiretroviral therapy (ART) and testing through the treat all approach as advised by the World Health Organization resulted in a decrease in the number of HIV infections [4]. In 2014, Eswatini achieved a 44% reduction of HIV infection from 23.1 new infections to 15.4 per 1,000 adults aged 15–49 years in 2018 [2]. The ART coverage among adults aged 15–49 years increased from 34% in 2010 to 74.1% in 2016 with a reduction of HIV incidence of 43.6% (95% CI: 39.71, 46.36) and HIV related deaths of 56.2% (95% CI: 54.06, 58.92) [5]. Pre-exposure and post-exposure prophylaxis have also been scaled up, to maximize prevention efforts for HIV infection in Eswatini [5]. Since 2009, voluntary medical male circumcision (VMMC) has been a priority in Eswatini. The prevalence of VMMC improved from 14% in 2008 [6] to 25% in 2014 [7]. Despite the several interventions being implemented HIV infection is still disconcertingly high in Eswatini, especially among women [2, 8–12].

Behaviors such as unprotected sexual intercourse, inconsistent condom use, poverty and unemployment, multiple sexual partners, substance abuse, and early sexual debut have been documented to sustain the transmission of HIV [13–15]. It has long been documented that behavioral patterns in sexual relationships play a significant role in the transmission of HIV/AIDS and STIs [13, 16–18]. Some public health experts believe that multiple concurrent partnerships and high partner acquisition are the main drivers of the persistent and high incidence of HIV in Sub-Saharan Africa (SSA) [19, 20]. Evidence suggests that multiple sexual partners during acute HIV infection are a driving force in heterosexually transmitted HIV epidemics in SSA, including Eswatini [6, 21–23]. Multiple sexual partnerships coupled with inconsistent condom use increase the risk of HIV acquisition [3, 23]. Women are biologically more at risk of contracting HIV and STIs than men and the risk of transmission from men to women is high compared to men [24, 25]. Developing countries have a high burden of STIs posing a major health burden for women aged 15–49 years who are more likely to be asymptomatic and have serious complications, such as pelvic inflammatory disease, leading to infertility and ectopic pregnancies [26]. Correspondingly, there is a need for a better understanding of the risk factors for multiple sexual partners among women in the context of Eswatini. Considerable research has explored the association of individual and household level factors on multiple sexual partners among women in a number of SSA countries [6, 17, 27, 28]. Very few attempts have been made to investigate community-level factors that influence the lifetime number of sexual partners, especially in Eswatini. This limitation is essential since the sexual behaviour of an individual is not purely dependent on individuals’ choices or circumstances but also determined by the aggregate level (social environment) factors in which individuals are nested in communities [1, 29]. Therefore, this study aimed to investigate the association between a range of individual and community variables on the lifetime number of sexual partners in Eswatini.

## Methods

### Data source

This study was a secondary analysis of data from the 2014 Eswatini Multiple Indicator Cluster Survey (MICS). The MICS is a global initiative designed to assist countries in collecting and analyzing data for monitoring the situation of children, women, and men in developing countries. It is a cross-sectional household survey conducted every three to five years to enable countries to monitor key indicators such as those related to health, education, and development. In the Eswatini Multiple Indicator Cluster Survey (EMICS), data was collected from a nationally representative sample of women using a standardized survey questionnaire through face-to-face interviews [7].

### Study design and sampling

The EMICS was representative and collected information on households, men, women, and children. The sampling frame of the enumeration was based on the 2007 Eswatini Population and Housing Census [30]. In the EMICS, a two-staged sampling technique was applied. First, enumeration areas (EAs), also known as the primary sampling units (PSUs), were selected. Second, households were selected stratified by the rural and urban residence and the four regions of the country, which are Manzini, Hhohho, Shiselweni, and Lubombo. A detailed description of the sampling approach can be found in the 2014 EMICS [7].

A total of 5001 women aged 15–49 years identified in the households were eligible for the survey interview. However, a total of 4,762 completed the survey (Table 1). We restricted our analysis to 2,832 women aged 15–49 years with complete data on their lifetime number of sexual partners and other characteristics. Data collection for the 2014 EMICS was conducted between July and November 2014.

**Table 1:** Household response rates and women response rates.

	Place of residence		
Households	Urban	Rural	Total
Households selected	1,351	3,860	5,211
Households interviewed	1,286	3,579	4,865
Household response rate	95.2%	95.7%	93.4%
**Women age 15–49**			
Number of eligible women	1,128	3,873	5,001
Number interviewed	1076	3686	4,762
Response rate	95.4%	95.2%	95.2

### Study variables

#### Outcome variable

The study outcome variable was the lifetime number of sexual partners. To get the women’s lifetime number of sexual partners, they were asked, “In total, with how many different people have you had sexual intercourse in your lifetime. Women provided a numerical answer.

#### Explanatory variables

*Individual-level factors*. Based on the literature [23, 29, 31–34], the individual and community level factors were coded as follows: age of the women categorized into seven age groups, 15–19, 20–24, 25–29, 30–34, 35–39, 40–44 and 45–49. Women's education level was categorized as no education, primary, secondary, high school, and tertiary. Marital status was stratified as current married/in union, formerly married/in union, or never married/in union. Age at first sexual intercourse was redefined as <15 years, 15–17 years, 18 years and older. Condom used at last sexual intercourse was stratified as yes and no. The household wealth index was redefined as poor, middle, and rich. The wealth index was used as a proxy for the standard of living in the households and used information about household amenities, such as housing materials, toilet or latrine access, phone ownership, agricultural land, and livestock, which are regularly collected in most household surveys such as the MICS [33]. The principal component analysis (PCA) was used to develop the household wealth index [35]. The community-level factors were the area of residence stratified as rural and urban, the region of residence stratified as Hhohho, Manzini, Shiselweni, and Lubombo, and the proportion of households classified under the poorest and poor wealth quantiles. The proportion of households classified under the poorest and poor wealth quantiles in the community was categorized as high and low [26, 36]. It was developed by aggregating the household wealth index at the PSU, which is a proxy for the community [26, 36].

### Statistical analysis

Stata 15 was used for the analysis, and weights were applied due to the hierarchal nature of the EMICS. Descriptive statistics in the form of frequencies and percentages were used to describe the sample against the explanatory variables of the study. The mean, with the standard errors along with the 95% confidence intervals was used to describe the distribution of the lifetime number of sexual partners against the covariates. The Poisson model could not be used for this analysis, due to over dispersion, had a conditional variance of 3.87 larger than the mean of 2.78 [37, 38]. To address the limitation of the Poisson model, the negative binomial regression was used as a preferred model [37, 39]. We, therefore, applied a two-level multilevel negative binomial regression model to report the measures of variation and fixed effects (measures of association) estimates of the model. Other studies have applied a similar method to model over dispersed count data [24, 26]. Four models were specified for the analysis: model 0 (empty model) included only the outcome variable, model 1 included only the individual factors; model 2 included only the community-level factors and model 3 included the individual and community-level factors in one model. We exponentiated the model coefficients and interpreted as a rate ratios (RRs) [25] with their 95% confidence intervals (CIs).

### Ethical considerations

The UNICEF team granted permission for the access and use of the EMICS dataset from http://mics.unicef.org/surveys. They are anonymous and do not allow the identification of participants. The data is publicly available and the authors had no special access privileges to the data and that other researchers will be able to access the data in the same manner as the authors.

## Results

The sample distribution, mean lifetime number of sexual partners, and bivariable results are presented in Table 2. The overall mean number of lifetime sexual partners was 2.78 (95% CI: 2.66, 2.91) among women. The mean number of lifetime sexual partners was lower among women aged 15–19 years and higher for those aged 20 years and older. The mean number of reported lifetime sexual partners was nearly equal by women education level and was higher among formerly married versus currently married. The mean number of reported lifetime sexual partners decreased with the age of sexual debut. For example, women who had sexual intercourse when they were 18 and older had a mean of 2.50 versus 3.47 among those aged less than 15 years and 3.04 among those aged 15–19 years. The mean number of reported lifetime sexual partners was higher among women that did not stay with their sons and daughters versus those that lived with their sons and daughters. The mean number of reported lifetime sexual partners was higher among women that resided in households that were classified as rich versus those that were poor. Women that lived in urban areas reported a higher mean number of reported lifetime sexual partners compared to those that resided in rural areas. The mean number of reported lifetime sexual partners was nearly equal across all four regions of residence, although the Manzini and Hhohho regions had slightly higher. Women that were from communities with a low proportion of households classified as poorest and poor quantiles had a higher mean number of sexual partners when compared to those from communities with a high proportion of households classified as poorest and poor quantiles.

**Table 2:** Individual characteristics by lifetime number of sexual partners.

Variables	n (%)	Mean (standard error)	95%CI	URRs (95%CI)
**Individual-level factors**				
**Lifetime number of sexual partners**	n = 2,832	2.78 (0.06)	2.66,2.91	
**Age**				
15–19	134 (4.4)	1.63 (0.10)	1.43,1.84	1
20–24	504 (17.7)	2.44 (2.17)	2.17,2.70	1.39 (1.25,1.55)*
25–29	588 (21.2)	2.91 (0.99)	2.72,3.10	1.74 (1.57,1.94)*
30–34	562 (21.4)	2.80 (0.12)	2.56,3.04	1.74 (1.57,1.94)*
35–39	433 (14.8)	3.16 (0.14)	2.89,3.43	1.90 (1.71,2.12)*
40–44	362 (12.3)	2.89 (0.12)	2,65,3.13	1.81 (1.61,2.02)*
45–49	249 (8.2)	2.92 (0.129)	2.67,3.18	1.78 (1.58,2.00)*
**Education level**				
No education	167 (5.1)	2.93 (0.26)	2.42,3.45	1
Primary	765 (24.9)	2.79 (0.91)	2.61,2.97	0.94 (0.84,1.04)
Secondary	968 (34.7)	2.84 (0.11)	2.63,3.05	0.89 (0.81,0.99)*
High school	651 (24.5)	2.65 (0.09)	2.47,2.84	0.82 (0.74,0.91)*
Tertiary	281 (10.9)	2.79 (0.17)	2.45,3.13	0.92 (0.82,1.04)
**Marital status**				
Currently married	1750 (60.4)	2.48 (0.078)	2.33,2.64	1
Formerly married	256 (10.5)	4.25 (0.20)	3.85,4.64	1.52 (1.43,1.63)*
Never married	826 (29.2)	2.87 (0.86)	2.70,3.04	1.06 (1.01,1.12)*
**Age at first sexual intercourse (in years)**				
<15	198 (6.5)	3.47 (0.24)	3.01,3.94	1
15–17	1175 (40.4)	3.04 (0.08)	2.88,3.19	0.87 (0.80,0.94)*
18 and older	1459 (53.1)	2.50 (0.08)	2.34,2.67	0.71 (0.65,0.77)*
**Condom used at last sexual intercourse**				
Yes	1508 (56.2)	2.99 (0.08)	2.84,3.14	1
No	1324 (43.8)	2.51 (0.07)	2.37,2.65	0.88 (0.83,0.92)*
**Lived with sons and daughters**				
Yes	2351 (80.9)	2.65 (0.06)	2.53,2.78	1
No	481 (19.1)	3.33 (0.12)	3.10,3.57	1.20 (1.13,1.28)*
**Household wealth index**				
Poor	1171 (34.0)	2.49 (0.07)	2.36,2.62	1
Middle	615 (19.6)	2.77 (0.10)	2.58,2.95	1.04 (0.97,1.10)
Rich	1046 (46.4)	3.00 (0.10)	2.80,3.20	1.09 (1.03,1.16)*
**Community factors**				
**Place of residence**				
Rural	2153 (65.4)	2.58 (0.05	2.48,268	1.21 (1.14,1.29)*
Urban	679 (34.6)	3.16 (0.13)	2.91,3.42	1
**Region**				
Hhohho	734 (23.5)	2.84 (0.08)	2.68,3.00	1
Manzini	776 (41.2)	2.94 (0.12)	2.70,3.18	0.98 (0.91,1.05)
Shiselweni	743 (16.6)	2.45 (0.92)	2.27,2.63	0.86 (0.79,0.93)*
Lubombo	579 (18.7)	2.65 (2.39)	2.39,2.91	0.91 (0.84,0.99)*
**Proportion of women from households classified as poorest and poor in the community**				
Low	1387 (57.8)	2.99 (0.09)	2.82,3.17	1
High	1445 (42.2)	2.49 (0.06)	2.36,2.62	0.85 (0.81,0.90)*

The bivariable results are also shown in Table 2. All the individual and community level factors were significantly associated with the reported lifetime number of sexual partners in the unadjusted models.

### Multivariable multilevel model results

The random and fixed effects results are shown in Table 3. After controlling for both individual and community factors (model 3), women’s age, marital status, age at first sexual intercourse, condom use at last sexual intercourse, and women lived with sons and daughters were associated with the reported lifetime number of sexual partners. Women aged 20–24 (ARR = 1.51, 95% CI:1.29, 1.76), 25–29 (ARR = 2.01, 95% CI:1.73, 2.34), 30–34 (ARR = 1.99, 95% CI: 1.70, 2.32), 35–39 (ARR = 2.17, 95% CI:1.85, 2.54), 40–44 (ARR = 2.16, 95% CI: 1.84, 2.54), and 45–49 (ARR = 2.10, 95% CI: 1.77, 2.48) reported more lifetime sexual partners compared to women aged 15–19 years. Those that were formerly married, ARR = 1.68 (95% CI:1.55, 1.81) and never married, ARR = 1.34 (95% CI: 1.26, 1.43) reported more lifetime sexual partners compared to currently married women. After controlling for other factors in the model, women that were aged 15–17 years and 18 and older at sexual debut reported fewer lifetime sexual partners (ARR = 0.86, 95% CI: 0.79, 0.95) and (ARR = 0.67, 95% CI: 0.61, 0.74) respectively compared to those that were aged less than 15 years. Women that did not use a condom at last sexual intercourse reported fewer lifetime sexual partners (ARR = 0.91, 95% CI: 0.86, 0.95) compared to those that used a condom at last sexual intercourse. Relative to women that lived with their sons and daughters, those that did not live with their sons and daughters reported more lifetime sexual partners (ARR = 1.12, 95% CI: 1.05, 1.19). At the community level, women that lived in the Shiselweni and Lubombo region reported fewer lifetime sexual partners (ARR = 0.87, 95% CI:0.80, 0.93) and (ARR = 0.90, 95% CI: 0.83, 0.97) respectively compared to those residents in the Hhohho region.

**Table 3:** Individual and community-level factors and lifetime number of sexual partners.

Fixed effects	Model 0	Model 1	Model 2	Model 3
Individual level factors	ARR (95%CI)	ARR (95%CI)	ARR (95%CI)	ARR (95%CI)
**Age**				
15–19		1		1
20–24		1.51 (1.29,1.76)*		1.51 (1.29,1.76)*
25–29		2.01 (1.73,2.35)*		2.01 (1.73,2.34)*
30–34		1.99 (1.71,2.33)*		1.99 (1.70,2.32)*
35–39		2.18 (1.86,2.55)*		2.17 (1.85,2.54)*
40–44		2.16 (1.84,2.54)*		2.16 (1.84,2.54)*
45–49		2.09 (1.77,2.48)*		2.10 (1.77,2.48)*
**Education level**				
No education		1		1
Primary		1.00 (0.90,1.12)		1.00 (0.90,1.12)
Secondary		1.01 (0.91,1.13)		1.02 (0.91,1.14)
High school		0.94 (0.84,1.06)		0.95 (0.84,1.07)
Tertiary		0.97 (0.84,1.11)		0.97 (0.85,1.11)
**Marital status**				
Currently married		1		1
Formerly married		1.68 (1.56,1.81)*		1.68 (1.55,1.81)*
Never married		1.34 (1.26,1.43)*		1.34 (1.26,1.43)*
**Age at first sexual intercourse (in years)**				
<15		1		1
15–17		0.87 (0.79,0.95)*		0.86 (0.79,0.95)*
18 and older		0.67 (0.61,0.74)*		0.67 (0.61,0.74)*
**Condom used at last sexual intercourse**				
Yes		1		1
No		0.90 (0.86,0.95)*		0.91 (0.86,0.95)*
**Lived with sons and daughters**				
Yes		1		1
No		1.12 (1.05,1.19)*		1.12 (1.05,1.19)*
**Household wealth index**				
Poor		1		1
Middle		1.08 (1.01,1.16)*		1.05 (0.98,1.13)
Rich		1.16 (1.08,1.24)*		1.07 (0.99,1.16)
**Community factors**				
**Place of residence**				
Rural			0.88 (0.81,0.96)*	0.95 (0.88,1.02)
Urban			1	1
**Region**				
Hhohho			1	1
Manzini			0.98 (0.90,1.06)	0.94 (0.87,1.01)
Shiselweni			0.88 (0.81,0.96)*	0.87 (0.80,0.93)*
Lubombo			0.93 (0.84,1.01)	0.90 (0.83,0.97)*
**Proportion of households classified as poorest and poor in the community**				
Low			1	1
High			0.92 (0.85,0.99)*	0.94 (0.88,1.01)
**Random effects**	Empty	Individual	Community	Full model
Community variance	0.039 (0.01)*	0.018 (0.05)*	0.027 (0.01)*	0.011 (0.005)*
Log-likelihood	-5372.04	-5116.22	-5351.45	-5103.95
PVC	Reference	61.97	31.72	71.43
AIC	10750.07	10274.4	10718.9	10259.9
N	2,832	2,832	2,832	2,832

Notes. SE-standard error, AIC-Akaike's information criterion, PCV-proportion change in variance, ARR–Adjusted rate ratio, CI: Confidence interval

* Significant at p-value <0.05.

Table 3 also showed the results of the random effects. There was a significant variation of the lifetime number of sexual partners (τ = 0.039, p<0.001) across communities (Model 0), justifying the use of the multilevel model in our analysis. The variance remained significant even after controlling for individual and community-level factors (τ = 0.111, p<0.004) (model 4). The results revealed that the variation of the lifetime number of sexual partners was a result of the composition of communities by individual and community-level factors. The model fit statistics revealed that the Akaike information criterion (AIC) reduced when combining individual and community level factors in one model, indicating that the final model fitted the data well when compared to the other models. Therefore, model 4 was chosen to predict the number of lifetime sexual partners among women.

## Discussion

We examined the determinants of the lifetime number of sexual partners using a nationally representative sample of women for a better understanding of the predictors of lifetime number of sexual partners. We found that the mean number of lifetime sexual partners was 2.78 among women. Our findings revealed that a majority of women have multiple sexual partners in Eswatini and a number of factors were associated with the practice. Our results are comparable to multicountry studies done in SSA and Africa in general, that found women to have a mean of 2 to 3 lifetime sexual partners [19, 40]. Other studies conducted in SSA found that multiple sexual partnerships predispose women to the transmission and acquisition of STIs including HIV/AIDS [27, 30, 40].

We found that at the individual level, maternal age, marital status, age at first sexual intercourse, condom used at last sexual intercourse, lived with sons and daughters were associated with lifetime number of sexual partners. At the community level, only the region of residence was associated with the lifetime number of sexual partners. Multiple sexual partnership presents a health risk for the transmission of HIV and sexually transmitted infections; hence the results shared light for the strengthening of health promotion and sexual behavior educational programs among women aged 15–49 in Eswatini.

Predictably, the age of the women was positively associated with the number of lifetime sexual partners. Our results are in agreement with the literature [19, 40, 41]. The plausible explanation could be that older women accumulate more sexual partners as they get older.

Similar to other studies, we found that women that were never married and formerly married reported more lifetime sexual partners when compared to married women [27, 42]. Numerous studies reported that being married is associated with a wide range of health benefits [34, 43, 44]. According to the marital resources model, individuals that are married have better financial support, experience less stress and mortality [45]. Married individuals may be more likely to practice healthy behaviors especially women and may motivate each other as a couple to stay loyal to one another [46]. Formerly married or never-married women may have freedom from marital obligations and also more likely to participate in sexual networks for their survival and economic reasons [19].

We found that women that engaged in sexual intercourse when they were aged less than 15 years reported more lifetime number of sexual partners. In South Africa, the youth that engaged in early sexual debut reported more lifetime sexual partners [47]. Early sexual intercourse predisposes individuals to numerous negative health outcomes including STIs and HIV [48]. This may be because women that engaged in early sexual debut have a longer sexual duration that can increase the risk for HIV transmission and STIs [49, 50].

Women who did not use a condom during their last sexual intercourse reported few lifetime sexual partners. A plausible explanation for the finding could be that in this study, the majority of the women were married (see Table 2) supporting the assertion that a majority of people use condoms during casual contact outside their ongoing relationships [51, 52]. People with multiple sexual partners turn to have a higher frequency of condom use than people with a single partner [52]. Our results contradict those of the prior literature [19, 20], that people with multiple sexual partners have a higher risk to contract HIV, and this raises questions about factors like condom use that may be important modifiers of HIV risk in the context of multiple partnerships.

Several studies found that people from poor households had a greater number of sexual partners than those from richer households [33, 53, 54]. Contrary other studies found that wealthier people have a higher risk of exposure to HIV due to concurrent and multiple sexual behaviors [55, 56]. We found no significant difference in the women number of sexual partners by household wealth status.

Of particular interest are the effects of community-level factors in predicting the reported number of sexual partners among women in Eswatini. Community-level factors are important to explain the disparities that exist among individuals in communities [57, 58]. We found that women that were residents in the Shiselweni and Lubombo region reported fewer sexual partners than those in the Hhohho region. The Lubombo and Shiselweni region are the poorest regions in Eswatini compared to Manzini and Hhohho [59, 60]. In traditional communities’ women are more likely to marry early which reduces exposure to many sexual partners outside marriage.

In Nigeria women that were residents in communities that had a low and middle socioeconomic status were less likely to have reported multiple sexual partners relative to those resident in communities with higher socioeconomic status [58]. We found no significant variation in the reported number of sexual partners by community poverty. The results imply that community poverty may not be a factor that predicts multiple partnerships in Eswatini, but potentially other community factors.

### Study strength and limitations

This study results should be interpreted with caution. Causality cannot be established between the factors and the lifetime number of factors due to its cross-sectional design. The lifetime number of sexual partners was obtained through self-response from the respondents, which can result to recall bias [61]. Social desirability bias cannot be ruled out as participants may have misreported their lifetime number of sexual partners, age, wealth, and education level. The use of the household wealth index to estimate the socioeconomic status could be critiqued; however, developing countries similar to Eswatini lack valid data on income and expenditure. This study is among the first to document the lifetime number of sexual partners applying multilevel modelling in Eswatini. The EMICS data used a multistage sampling design and sampling weights to increase the representation of the sample to the total population of women aged 15–49 years in Eswatini.

## Conclusion

Applying the multilevel methodology, the study demonstrated that women aged 20 and older, formerly and never married, had sexual debut when they were less than 15 years reported more lifetime sexual partners. Fewer lifetime sexual partners were reported by women that did not use a condom during their last sexual encounter, lived with their sons and daughters, and were residents in the Shiselweni and Lubombo regions. Furthermore, a significant variation of the risk of the reported lifetime number of sexual partners was observed at the community level. The study has implications for policy, that behavioral change and sexual behavior strategies and programs should also target the social context independent of the individual factors. Sexual programs and health promotion education should go beyond the individualistic model to operate at the community level. Interventions that aim to influence early sexual debut among the youth could potentially reduce the number of lifetime sexual partners, which might reduce the risk of HIV acquisition if it concurs with other protective behaviors, such as condom use.
